# Evidence for a Prepore Stage in the Action of *Clostridium perfringens* Epsilon Toxin

**DOI:** 10.1371/journal.pone.0022053

**Published:** 2011-07-11

**Authors:** Susan L. Robertson, Jihong Li, Francisco A. Uzal, Bruce A. McClane

**Affiliations:** 1 Department of Microbiology and Molecular Genetics, University of Pittsburgh School of Medicine, Pittsburgh, Pennsylvania, United States of America; 2 California Animal Health and Food Safety Laboratory System, School of Veterinary Medicine, University of California Davis, Davis, California, United States of America; Columbia University, United States of America

## Abstract

*Clostridium perfringens* epsilon toxin (ETX) rapidly kills MDCK II cells at 37°C, but not 4°C. The current study shows that, in MDCK II cells, ETX binds and forms an oligomeric complex equally well at 37°C and 4°C but only forms a pore at 37°C. However, the complex formed in MDCK cells treated with ETX at 4°C has the potential to form an active pore, since shifting those cells to 37°C results in rapid cytotoxicity. Those results suggested that the block in pore formation at 4°C involves temperature-related trapping of ETX in a prepore intermediate on the MDCK II cell plasma membrane surface. Evidence supporting this hypothesis was obtained when the ETX complex in MDCK II cells was shown to be more susceptible to pronase degradation when formed at 4°C vs. 37°C; this result is consistent with ETX complex formed at 4°C remaining present in an exposed prepore on the membrane surface, while the ETX prepore complex formed at 37°C is unaccessible to pronase because it has inserted into the plasma membrane to form an active pore. In addition, the ETX complex rapidly dissociated from MDCK II cells at 4°C, but not 37°C; this result is consistent with the ETX complex being resistant to dissociation at 37°C because it has inserted into membranes, while the ETX prepore readily dissociates from cells at 4°C because it remains on the membrane surface. These results support the identification of a prepore stage in ETX action and suggest a revised model for ETX cytotoxicity, i) ETX binds to an unidentified receptor, ii) ETX oligomerizes into a prepore on the membrane surface, and iii) the prepore inserts into membranes, in a temperature-sensitive manner, to form an active pore.

## Introduction

*Clostridium perfringens* epsilon toxin (ETX) is the third most potent of all clostridial toxins [1], [2], [3], [4], thus earning it a listing as a CDC class B select toxin. ETX is only produced by type B and D isolates of *C. perfringens*, which cause fatal enterotoxemias in several livestock species [3], [4]. Those type B and D enterotoxemias develop when ETX is produced in the intestines and then absorbed into the circulation, allowing the toxin to target internal organs outside of the gastrointestinal tract. ETX then causes severe, often-fatal neurologic disturbances and edema in many internal organs, at least in part, by damaging vascular endothelial cells [3], [5], [6].

The ETX-encoding gene (*etx)* is carried by large plasmids, some of which share homology with the enterotoxin-encoding plasmids found in type A strains [7]. ETX is produced during vegetative growth, rather than by sporulating cells. It is then secreted, initially as a binding capable (but inactive) prototoxin of 311 amino acids (32.7 kDa) [8]. The ETX prototoxin can be proteolytically-activated by removal of 11-13 N-terminal amino acids and 22–29 C-terminal amino acids, with the size of the cleavage dependent upon the protease used [1]. Proteases capable of activating ETX include *C. perfringens* lambda toxin, as well as intestinal proteases such as trypsin or chymotrypsin [9]. Activation of the toxin *in vivo* is probably mediated mainly by the intestinal proteases present in the gastrointestinal tract.

Activated ETX is a pore-forming toxin that shares structural similarities with another pore-forming toxin (PFT) named aerolysin [10]. However, ETX is about 100-fold more potent than aerolysin at killing sensitive mammalian cells [11], reportedly via a necrotic process [10]. A channel-forming domain has been identified in ETX that resides between residues 151–180 [12]. This same ETX region also contains one or more neutralizing epitopes [13]. ETX regions mediating other functions have not yet been conclusively mapped.

It is generally accepted that ETX action on sensitive cells begins with the binding of this toxin to a still unidentified protein receptor. Distribution of the ETX receptor is apparently restricted to certain organs, including the brain, the lungs and the kidneys [14], [15], [16], [17], [18]. Similarly, ETX can affect only a few cell culture lines. Those ETX-sensitive cell lines include Madin–Darby Canine Kidney (MDCK) II cells, which are commonly used as an *in vitro* model to study the molecular action of ETX [2], [19], [20], [21], [22], [23]. In MDCK II cells, the toxin uses lipid rafts to form a large heptameric complex that is SDS-resistant and has an apparent size of ∼155 kDa [24]. Substantial evidence suggests that this ETX complex corresponds to a general diffusion pore permeable to molecules up to ∼1 kDa [12], [21]. This ETX pore mediates the release of K^+^ from, and influx of Na^+^ and Cl^−^ into, MDCK II cells [2], [25], [26].

Comparing *in vitro* studies performed at various temperatures often provide valuable insights into a toxin's mechanism of action [27], [28], [29]. Since the pioneering work by Petit et al. [2], it has been appreciated that ETX does not kill MDCK II cells at 4°C, despite MDCK II cells being one of the most ETX-sensitive cell lines at 37°C [2]. That study also reported that ETX can still bind and form a large complex at 4°C [2], although opposite conclusions have also been reported using a biologically-active ETX fusion protein [30]. Furthermore, no study has yet conducted a systematic step-by-step quantitative comparison of the occurrence of each step in ETX action at 4°C vs. 37°C.

Therefore, the current study quantitatively compared the effects of temperature differences on each known step in ETX action against MDCK II cells. By studying the nature of the low temperature blockage of ETX action, these analyses have provided the first experimental evidence for a prepore step in ETX action.

## Material and Methods

### Toxin

Epsilon prototoxin was purified from overnight cultures of *Clostridium perfringens* type D (NCTC 8346) as described previously [31], [32]. Briefly, a starter culture of strain NCTC 8346 was grown overnight at 37°C in fluid thioglycolate broth (Difco). That starter culture was then used to inoculate 100 ml of TGY (3% tryptic soy broth [Becton-Dickinson]; 2% glucose [Sigma Aldrich], 1% yeast extract [Becton-Dickinson], 0.1% sodium thioglycolate [Sigma Aldrich]). After 16–18 h of growth at 37°C, the TGY culture was added to 3.5 L of fresh TGY and grown for 12 h at 37°C. Following that inoculation, the culture was centrifuged and the supernatant was then incubated at 4°C for 1 h with ammonium sulfate before centrifugation. The precipitated material was dissolved in phosphate buffer (0.1M KH_2_PO_4_, 0.1 M Na_2_HPO_4_, pH 7.3) and dialyzed against the same phosphate buffer overnight at 4°C. The dialyzed sample was then applied to a DEAE cellulose column and fractions were collected, dialyzed and analyzed by electrophoresis or Western Blot and quantified by Lowery [33]. Those analyses showed the purified prototoxin preparation contained a single protein of ∼32 kDa.

The purified prototoxin was fluorescently labeled using an AlexaFluor 488 protein labeling kit (Invitrogen), following methods described by the manufacturer. Fluorescently-labeled toxin will be referred to as AF488-ETX throughout the manuscript. AF488-ETX and ETX had similar cytotoxicity for MDCK II cells, as assessed using the Live/Dead cell assay (described below).

Prior to use in assays as active ETX, aliquots of the prototoxin (whether labeled or not) were activated by incubation with 12.5 µg of trypsin (Sigma)/µg toxin for 1 h at 37°C. Following this activation, trypsin inhibitor (Sigma) was added to remove trypsin activity. Therefore, all cytotoxicity/morphology assays used the trypsin/trypsin inhibitor mix as a control, as well as a non-trypsin-treated prototoxin control.

### Cell culture and animal tissue

Madin-Darby Canine Kidney (MDCK II) epithelial cells [34] were routinely cultured in a 50/50 (v/v) mix of Dulbecco's Modified Eagle's Medium (DMEM, Sigma) and Nutrient Mixture F12 HAM (Sigma), supplemented with 3% fetal bovine serum, 100 units/ml penicillin, 100 µg/ml streptomycin, and 1% glutamine. Cells were maintained at 37°C in 5% atmospheric CO_2_ and grown until confluency in 75 cm^2^ flasks before seeding for experimentation.

A 0.05 g aliquot of Balb/C mouse brain or kidney tissue was weighed out and homogenized in 0.5 ml of PBS supplemented with 0.4 M NaCl, 0.05% Tween-20, 0.5% BSA and 10 mM EDTA and Protease inhibitors (Roche). The tissue homogenates were then stored at −80°C until needed.

### Cell Morphology and Cytotoxicity

To visualize ETX-induced morphologic damage, confluent MDCK II cells in 100 mm^2^ cell culture dishes (Corning) were incubated in the presence or absence of 10 µg of ETX for 60 min at 4°C or 37°C. MDCK II cells treated with ETX at 4°C were also assessed for morphological damage after a shift to 37°C. This was performed as described earlier except that, following incubation with ETX at 4°C for 60 min, the cells were washed and then warmed to 37°C. After a 1 h incubation, pictures were taken of the control cells, prototoxin-treated cells, and ETX-treated cells using a Canon Powershot G5 fitted to the Zeiss Axiovert 25 microscope [35].

MDCK II cell cytotoxicity was measured using the Live/Dead viability/cytotoxicity kit for mammalian cells (Invitrogen), as previously described [36], or the LDH Cytotoxicity Detection Kit (Roche). Briefly for the Live/Dead assay, confluent MDCK II cells cultured in 96 well plates (Corning) were treated with 10 µg of ETX or prototoxin for 60 min at 4°C or 37°C before cytotoxicity was measured. For the LDH assay, confluent MDCK II cultures grown in 100 mm^2^ plates were treated with 10 µg of ETX for 60 min at 4°C or 37°C. Alternatively, some MDCK II cells were treated with 10 µg of ETX for 60 min at 4°C; after washing, those cells were warmed to 37°C for 1 h. Supernatant was removed from all treated cultures and then transferred to individual wells of a 96 well plate before the reagents from the LDH kit were added and cytotoxicity was measured using an iMark microplate reader (BIO-RAD), with an absorbance wavelength of 490 nm and a reference wavelength of 600 nm. The data was recorded and analysed using the Microplate manager software version 6.1.

### Analysis of ETX complex formation at 4°C versus 37°C in mouse brain or kidney homogenates

To assess ETX complex formation in brain and kidney tissues obtained from healthy untreated mice, 100 µl of homogenized tissue (kidney or brain), prepared as described earlier, were incubated with 10 µg of AF488-ETX for 60 min with gentle rocking at either 4°C or 37°C. Upon completion of this incubation, 100 µl of 2 X loading buffer (50 mM Tris pH 6.8, 2% SDS, 10% Glycerol and 0.05% bromophenol blue) was added to the sample. The ETX-treated mouse kidney or brain tissue samples (or ETX-treated MDCK II cell samples for comparison) were then electrophoresed on an 8% polyacrylamide gel containing SDS. The resultant gel was imaged using a Typhoon 9400 variable mode imager (Amersham Biosciences), with fluorescence emission set to detect the Alexafluor 488 label using the green laser with wavelength 532 nm or, for detection of the molecular weight markers, the red laser was used with a wavelength of 633 nm. Complex formation was then quantified using Imagequant version 5.2 (Molecular Dynamics). Upon completion of the first fluorescent complex scan, separated proteins on the gel were transferred to nitrocellulose membrane and then probed with rabbit polyclonal anti-ETX antibody (catalog number NR-865 from BEI resources, Manassas, VA) to ensure that this was indeed the ETX-containing complex (data not shown).

### Pronase digestion susceptibility of ETX complex formed at 4°C vs. 37°C

The relative susceptibility to pronase digestion of ETX complex formed in MDCK II cells at 4°C vs. 37°C was analyzed using an assay adapted from previously published methods [37]. Confluent MDCK II cells grown in a 6 well plate were treated with 10 µg of AF488-ETX for 60 min at either 4°C or 37°C. Those MDCK II cells were harvested and washed; half of this sample was left intact, while the other half was lysed in RIPA buffer (50 mM Tris, 150 mM NaCl, 0.1% SDS, 0.5% C_24_H_39_NaO_4_, 1% Triton-X100 and protease inhibitors added before use). Both sets of samples were incubated with 6, 60 or 600 ng of pronase (Sigma) for 60 min at 4°C. At that time the intact MDCK II cells were washed with HBSS and resuspended in 50 µl of HBSS and 2x loading buffer, whereas 2x loading buffer was added in equal volume to the MDCK II cell lysates. Samples were electrophoresed side-by-side on an 8% polyacrylamide gel containing SDS in order to compare the pronase digestion susceptibility of ETX complexes in whole cell vs. lysates at the two different temperatures. Fluorescent gels were imaged as described earlier.

### Measurement of ETX pore formation in MDCK II cells

A ^86^rubidium release assay is often used to evaluate toxin-induced pore formation that leads to mammalian plasma membrane permeability changes [35]. Therefore this assay was employed to assess the ability of ETX to form pores in MDCK II cells. Confluent MDCK II cells grown in a 24 well plate (Corning) were radiolabeled for 3 h at 37°C in HBSS containing 4 µCi of ^86^RbCl (Perkin-Elmer) per well. After radiolabeling, the cells were washed twice and treated, at 4°C or 37°C, for 5, 10 or 20 min with 10 µg of ETX. Culture supernatants were collected and counted (cpm) using a Beckman gamma counter. After subtraction of spontaneous (background) ^86^Rb release, data were plotted as the percentage of maximal ^86^Rb release that was specifically induced by each ETX treatment, determined as published previously [35].

### Dissociation of ETX complex at 4°C or 37°C

MDCK II cells were washed and treated with 10 µg of AF488-ETX for 60 min at either 4°C or 37°C. The supernatants containing unbound toxin were removed and the cultures were further incubated at the toxin treatment temperature for 0, 15, 30 or 60 min. After that incubation, adherent cells were harvested by gentle scrapping and combined with nonadherent cells floating in the supernatant. These collected cells were then washed three times and resuspended in 50 µl of PBS buffer with 1 µl of benzonase. SDS loading buffer (2X) was added for lysis and the samples were electrophoresed using the conditions described earlier for complex analysis.

Additionally, the stability of solubilized ETX complex that had been formed in MDCK II cells at 4°C vs. 37°C was compared in the presence of 10% SDS. MDCK II cells were treated with 10 µg of AF488-ETX for 60 min at either 4°C or 37°C. After that treatment, the cells were washed and incubated for desired time periods. At the completion of the incubation time, the cells were harvested and washed twice in HBSS before electrophoresis on 8% polyacrylamide gels containing 10% SDS to determine the stability of the complex that had been formed at both temperatures.

### Ethics Statement

This study was carried out in strict accordance with the recommendations in the Guide for the Care and Use of Laboratory Animals of the National Institutes of Health. The protocol was approved by the Animal Care and Use Committee of the University of California, Davis (Permit Number: 16258). All efforts were made to minimize suffering of mice. The MDCK II cell line used in this study is not a de novo cell line and was obtained from Dr. James Anderson, who previously published using this well-established cell line [34].

## Results

### Comparison of morphologic damage and cytotoxicity in MDCK II cells treated with ETX at 4°C vs. 37°C

**Figure 1:**
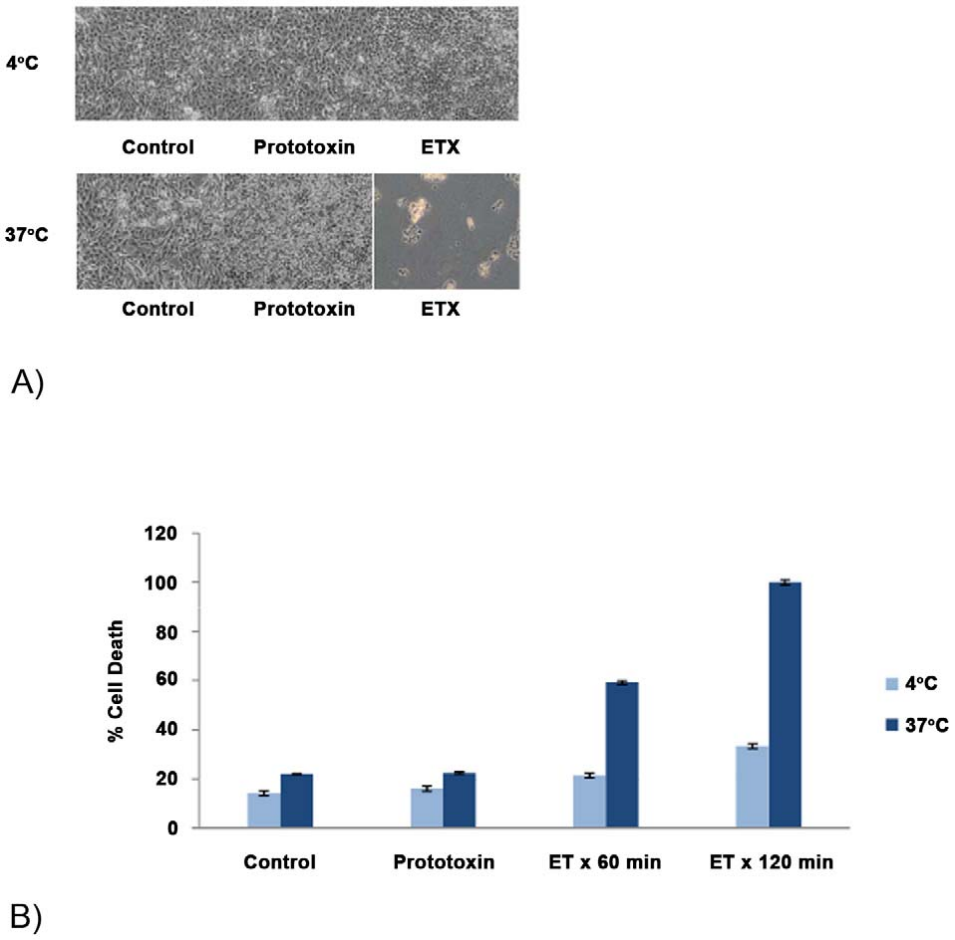
Temperature effects on ETX-induced MDCK II cell morphologic damage. A) Cell morphology. MDCK II cells were treated with 10 µg of ETX for 60 min at 4°C or 37°C and photographed. B) Cytotoxicity. MDCK II cells were treated with 10 µg of ETX for 60 or 120 min at 4°C or 37°C and cytotoxicity was then measured using the Live/Dead viability/cytotoxicity kit for mammalian cells. Shown is the average for 3 experiments.

It was previously reported that both native ETX and an ETX-green fluorescent protein (GFP) fusion protein are cytotoxic for MDCK II cells at 37°C, but not at 4°C [2], [11]. The current study first confirmed those conclusions by treating MDCK II cells with ETX for 60 min. Both ETX-induced morphologic damage and cytotoxicity were readily detectable within 60 min when the treatment temperature was 37°C (Fig. 1A and 1B). In contrast, after 60 min of similar ETX treatment at 4°C, the MDCK II cells did not exhibit either morphological damage (Fig. 1A) or cytotoxicity (Fig. 1B), as detected by a Live/Dead assay. Even after 2 h of ETX treatment at 4°C only a small increase in cytotoxicity was detected over control (no ETX treatment) levels.

### Comparison of AF488-ETX binding and complex formation levels in MDCK II cells treated with ETX at 4°C vs. 37°C

**Figure 2:**
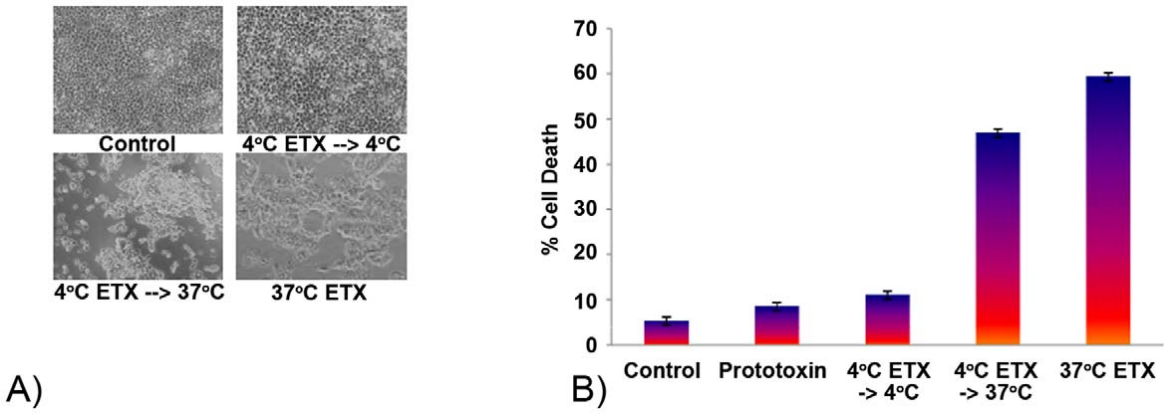
MDCK II cells treated with ETX at 4°C are efficiently killed when shifted to 37°C. A) Cell morphology. MDCK II cells were treated with 10 µg of ETX for, i) 60 min at 4°C, followed by washing and further incubation at 4°C, ii) 60 min at 4°, followed by washing and incubation at 37°C for 1 h or iii) 60 min at 37°C. B) Cell cytotoxicity. Cell death in the cultures described in panel A was measured by the LDH assay. Shown is the average from 3 experiments.

A possible explanation for the loss of cytotoxicity at 4°C, as observed in Fig. 1, might be that ETX cannot bind or form complexes at low temperature. Whether ETX binds and forms complexes in MDCK II cells at 4°C has remained somewhat unsettled in the literature. Petit et al. reported detection of ETX complexes in MDCK II cells treated with ETX at 4°C [2]. However, Soler-Jover et al. [30] reported that when MDCK II cells were treated at 4°C with an ETX-GFP fusion protein (which is cytotoxic at 37°C), washed, and then warmed to 37°C, no cytotoxicity developed [30]. Since that result supported a possible impairment of ETX binding at 4°C, the current study repeated this experiment by treating MDCK II cells at 4°C with native ETX instead of using an ETX-GFP fusion protein. The results obtained clearly indicated that ETX had bound to functional ETX receptors at 4°C, i.e., strong morphologic damage (Fig. 2A) and cytotoxicity (Fig. 2B) developed when those cells were washed and then warmed to 37°C for 1 h. Notably, the levels of morphologic damage and cytotoxicity that occurred in these temperature-shifted cells was nearly that observed using the same ETX treatment constantly at 37°C for 1 h.

**Figure 3:**
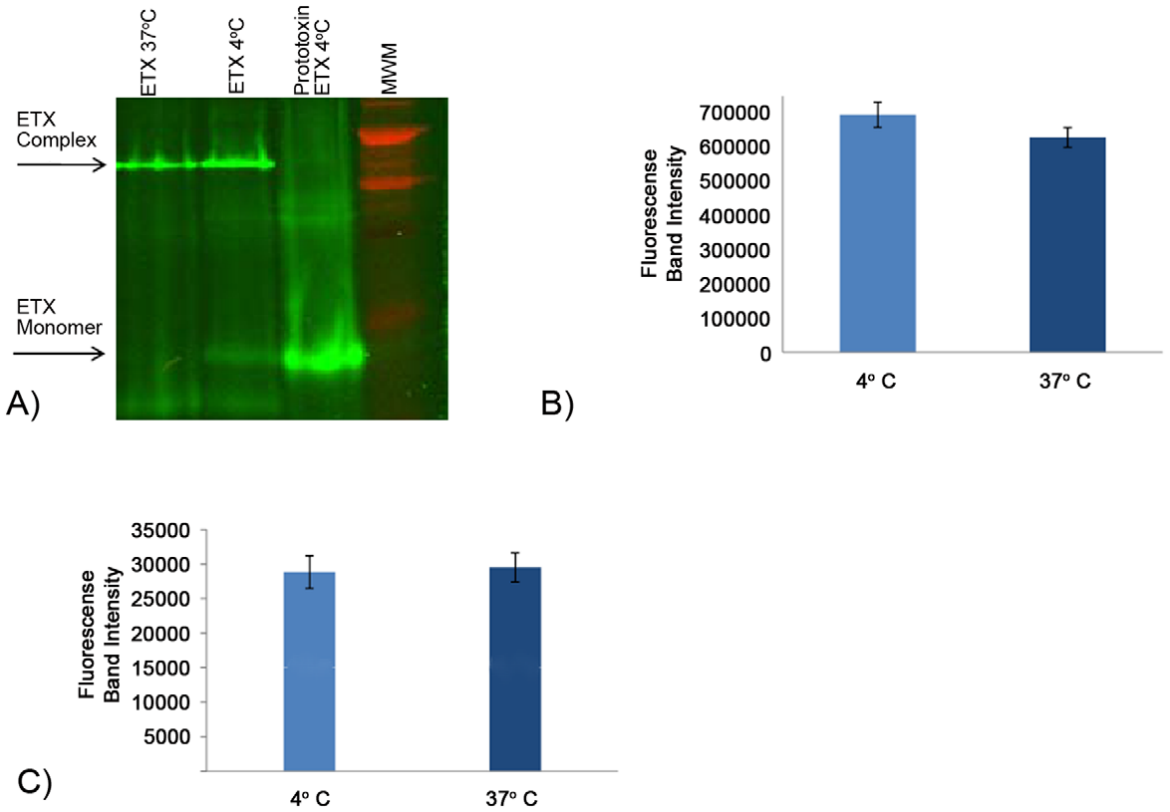
Analysis of temperature effects on ETX binding and complex formation in MDCK II cells. A) ETX complex formation. MDCK II cells were treated with 10 µg of AF448-ETX for 60 min at 4°C or 37°C and then electrophoresed and quantitatively imaged (see Materials and Methods). Arrows indicate, as specified, migration of the ∼155 kDa ETX complex or the ∼30 kDa ETX monomer. B) Quantitative analysis of ETX binding at 4°C vs. 37°C. MDCK II cells were treated with 10 µg of AF488-ETX for 60 min at 4°C or 37°C. After electrophoresis of cell lysates, total AF488-ETX present in the gel i.e. AF488-ETX bound in complex or as monomer, was quantified by fluorescence scanning. Shown are the average results from 4 gel scans. C) Quantitative analysis of ETX complex formation. MDCK II cells were treated with 10 µg of ETX for 60 min at 4°C or 37°C. The amount of fluorescence specifically present in gel regions containing AF488-ETX bound in the ETX complex was determined. Shown are the mean values from 4 experiments.

Having obtained evidence supporting ETX binding at 4°C, the current study next conducted the first head-to-head quantitative comparison of ETX binding levels and complex formation levels at 4°C vs. 37°C. When quantitative fluorescence analysis was performed on gels containing samples of MDCK II cells treated with AF488-ETX at either 4°C or 37°C (Fig. 3A), similar amounts of bound toxin were detected at either 4°C or 37°C (Fig. 3B). This analysis further determined that, i) ∼70–90% of the AF488-ETX bound to MDCK II cells was localized in the ETX complex and ii) nearly equivalent amounts of ETX complex had formed in MDCK II cells at both temperatures (Fig. 3C).

### Temperature effects on ETX complex formation in mouse tissues

Mouse kidney and brain are the major organs that bind ETX at 37°C and are considered important ETX targets *in vivo* [11], [15], [38], [39]. While ETX complex formation in brain synaptomosomal membranes has been previously shown at 37°C [24], ETX complex formation in kidney tissue preparations has not yet been demonstrated, to our knowledge. Furthermore, ETX complex formation has not been compared at 4°C vs. 37°C using preparations from either brain or kidney.

**Figure 4:**
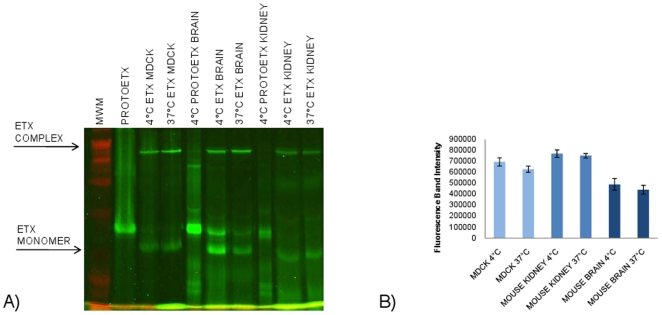
Analysis of ETX complex formation in mouse tissues at 37°C and 4°C. A) ETX complex formation in MDCK II cells or homogenized mouse brain or kidney tissue. A 10 µg aliquot of ETX was incubated with MDCK II cells or homogenized tissue for 60 min at 4°C or 37°C, as indicated. Those samples were then electrophoresed as described above. Arrows depict, as specified, migration of the ∼155 kDa ETX complex or the ∼30 kDa ETX monomer. B) Quantitative analysis of complex formation in MDCK II cells and mouse tissue. Fluorescence quantification of ETX complex levels demonstrated that the amount of complex formed in MDCK II cells or homogenized mouse tissues was not temperature-sensitive. Shown is the average from 3 experiments.

Therefore, to help confirm that the Fig. 3 results also apply to natural ETX target tissues, an experiment examined ETX complex formation in homogenized mouse brain and kidney tissues both at 4°C and 37°C. Results from analyses first showed that AF488-ETX forms a complex in homogenized kidney tissue that is similarly-sized as the ETX complex formed in either homogenized brain tissue or MDCK II cells (Fig. 4A). Furthermore, this experiment detected no significant temperature-related differences in the amounts of AF488-ETX complex formed in either brain or kidney homogenates (Fig. 4B).

### Comparison of ETX pore formation in MDCK II cells at 4°C vs. 37°C

Pore formation is considered to be important when ETX kills MDCK II cells, with the ETX complex corresponding to this pore [2], [12], [25]. Since the Figs. 1, 2, 3, and 4 results indicated that ETX performs the first two steps in its action, i.e., binding and complex formation, equally well at 4°C vs. 37°C, the low temperature block in ETX cytotoxicity observed in Fig. 1 might be attributable to an inhibition of pore formation at 4°C. To our knowledge, whether ETX can form functional pores in MDCK II cells at low temperature has not yet been assessed.

**Figure 5:**
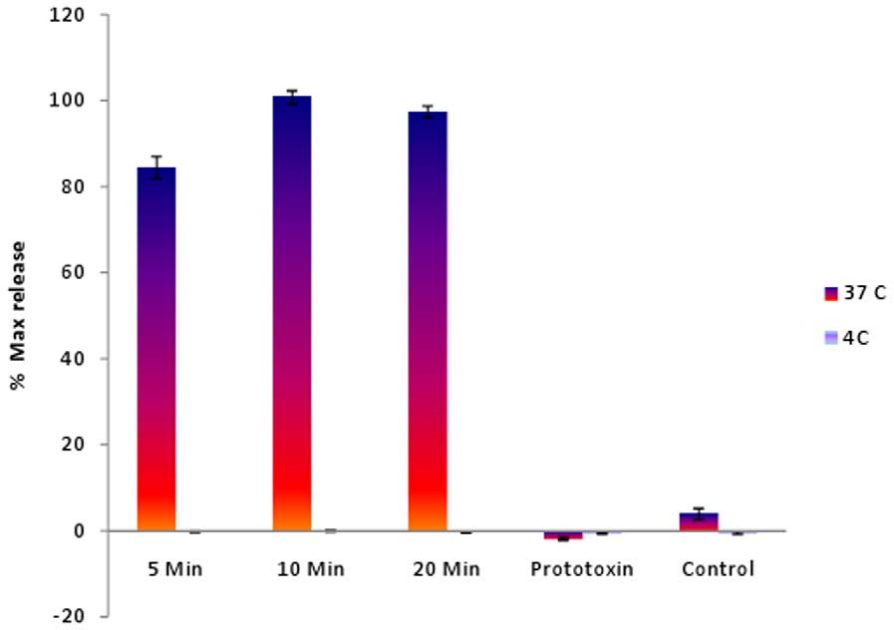
ETX effects on ^86^Rb-release from MDCK II cells at 4°C or 37°C. MDCK II cells were labeled with ^86^Rb for 3 h at 37°C, followed by incubation with 10 µg of ETX for 5, 10 or 20 min at 4°C or 37°C. Culture supernatants were then collected and cpm were measured using a Packard Cobra II gamma counter. After correction for spontaneous release, data were plotted as a percentage of maximal release with the error bars representing SE. Shown are the mean results from three experiments, with each experiment using duplicate samples.

Formation of the ETX pore was previously shown to cause rapid efflux of cytosolic potassium from MDCK II cells [2]. Therefore, to evaluate whether ETX pore formation is temperature-sensitive, the current study compared the effects of temperature on ETX-induced release of ^86^Rb, a potassium transport analogue, from MDCK II cells. When ^86^Rb-radiolabeled MDCK II cells were treated with 10 µg of ETX at 4°C, no ^86^Rb release over background levels was measured. However, significant ^86^Rb release over background levels was detected within 5 min of similar ETX treatment at 37°C. After correction for spontaneous background release, this ETX-induced ^86^Rb release reached ∼100% within 10 min at 37°C (Fig. 5).

### Investigating the mechanistic basis for the inhibition of ETX pore formation at 4°C

The Fig. 5 results clearly demonstrated that ETX pore formation is blocked at 4°C, offering an explanation for the observed loss of ETX-induced cytotoxicity at low temperature. The ability of the ETX complex to form at both 37°C and 4°C (Fig. 2), coupled with the absence of pore formation at 4°C (Fig. 5), suggested that ETX action might involve assembly of a “pre-pore” ETX complex on membrane surfaces prior to temperature-sensitive insertion of this complex into lipid bilayers to form a functional membrane pore. The current study explored this possibility using two experiments that have previously provided evidence for a prepore step in the action of *C. perfringens* enterotoxin, another PFT [37], [40].

**Figure 6:**
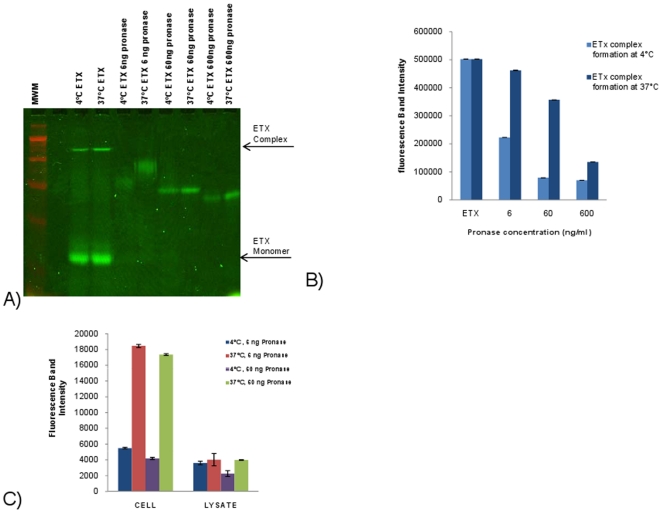
Comparison of pronase susceptibility of ETX complex formed in MDCK II cells at 4°C or 37°C. A) Comparison of pronase digestion of ETX complex in intact MDCK II cells at 4°C or 37°C. MDCK II cells were treated as described above with the exception that, following treatment, some MDCK II cells were lysed and then subjected to pronase digestion. Arrows denote, as specified, migration of the ∼155 kDa ETX complex or the ∼30 kDa ETX monomer. B) Fluorescence quantification of pronase dose effects on digestion of ETX complexes in intact MDCK II cells formed at 4°C vs. 37°C. C) Fluorescence quantification of pronase digestion of ETX complexes, formed at either 37°C or 4°C, in intact MDCK II cells vs. MDCK II cell lysates. This analysis showed that ETX complexes formed at either 4°C or 37°C are extensively digested when present in cell lysates but only the ETX complexes formed at 4°C are pronase-digested when using intact cells. These results suggest that in intact cells the ETX complex formed at 37°C has inserted into the membrane. Results shown are the average of 3 experiments.

The first experiment evaluated whether the ETX complexes present in intact MDCK II cells exhibit differing susceptibility to pronase digestion when formed at 4°C vs. 37°C. The premise of this experiment is that, i) at 37°C, membranes may provide an inserted ETX complex with some protection from digestion by externally-applied pronase, while ii) the ETX complex formed at 4°C should show greater sensitivity to similar pronase challenge if it remains exposed on the membrane surface in a prepore state. This experiment was performed using MDCK II cells containing AF488-ETX complex formed at 4°C or 37°C, washed at 4°C, and then treated with pronase at 4°C. As shown in Fig. 6A and 6B, more pronase digestion was noted for the ETX complex using intact MDCK II cells containing complex formed at 4°C vs. 37°C, This result is consistent with the ETX complex formed at 4°C remaining exposed as a prepore on the membrane surface and the ETX complex formed at 37°C having inserted into membranes.

An alternative interpretation for the Fig. 6A and 6B results could be that the ETX complex formed at 37°C is simply inherently more resistant against pronase digestion than the ETX complex formed at 4°C because of some significant conformational change. Arguing against this possibility are the results obtained when pronase was added to solubilized ETX-treated MDCK II cells. In this experiment, the solubilized ETX complexes formed at 4°C or 37°C were both pronase-digested at 4°C to a similar extent (Fig. 6C). Since ETX does not internalize into MDCK II cells, the Fig. 6 results collectively suggest that plasma membrane insertion explains the protection against proteolysis observed for intact MDCK II cells containing ETX complex formed at 37°C (Fig. 6A and 6B). By extension, these results support the hypothesis that ETX added to cells at 4°C becomes trapped in a prepore on the membrane surface because the prepore is blocked for insertion.

**Figure 7:**
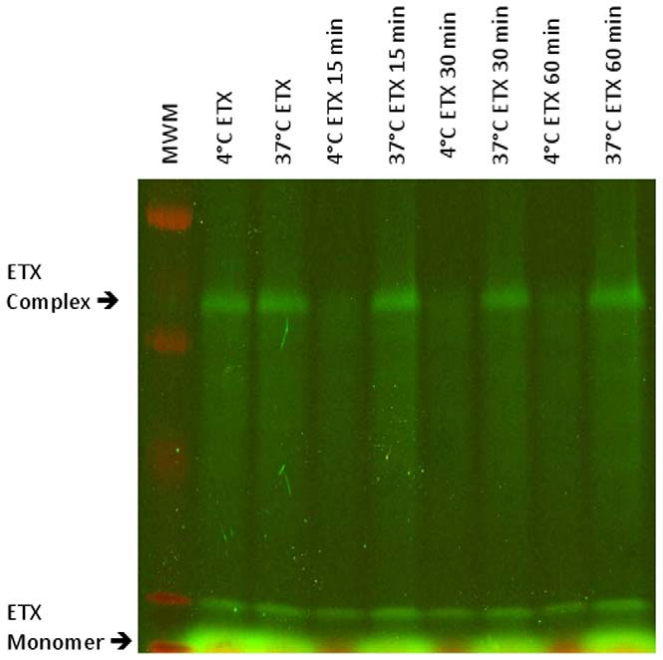
Temperature effects on the dissociation of ETX complex from MDCK II cells. MDCK II cells were treated with 10 µg of ETX for 60 min at 4°C or 37°C. Dissociation of the complex formed in those cells was then assessed over 15, 30 and 60 min at the same temperature used for toxin treatment. After washing, samples were subjected to SDS-PAGE and complex was then visualized using the Typhoon 9400 variable mode imager. Arrows depict, as specified, migration of the ∼155 kDa ETX complex or the ∼30 kDa ETX monomer.

To further test the hypothesis that ETX becomes immobilized in a prepore complex on the membrane surface at 4°C, the dissociation of bound AF488-ETX from MDCK II cells was compared after treatment with the labeled toxin at 4°C or 37°C (Fig. 7). The premise of this experiment is that, relative to dissociation of a prepore localized on the membrane surface at 4°C, insertion of the complex into membranes to form pores at 37°C should reduce ETX complex dissociation from MDCK II cells. For this experiment, MDCK II cells were treated with AF488-ETX at 4°C or 37°C, washed and dissociation of ETX from those washed cells or membrane fractions at the toxin treatment temperature was then examined over time. This analysis revealed that substantially less dissociation of bound ETX complex occurs from MDCK II cells when toxin treatments and subsequent incubations are performed at 37°C versus 4°C (Fig. 7). More dissociation of bound AF488-ETX complex was also observed from isolated membranes of MDCK II cells treated with toxin at 4°C vs. 37°C (data not shown). There was also little or no ETX monomer remaining in cells that had been treated with ETX at 4°C, washed and then incubated at 4°C for 15 min or more. However, cell-associated ETX monomer remained present in samples treated with toxin at 37°C, washed, and then incubated for 15 min or more. This cell-associated monomer in the 37°C samples might reflect partial breakdown of the ETX complex to monomers during the washing and extraction procedure. The stability of the ETX complex in membranes deserves future analysis.

### Evidence for conformational differences in ETX complex formed at 4°C vs. 37°C

**Figure 8:**
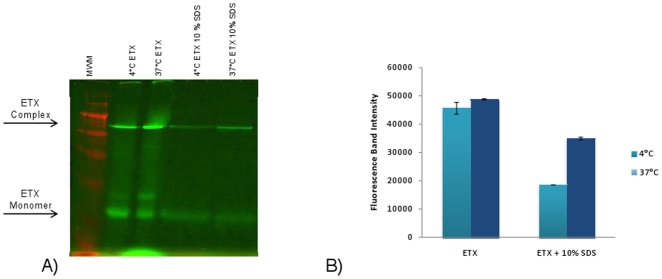
Stability of ETX complex formed at either 4°C or 37°C in the presence of high SDS concentrations. A) SDS disruption of ETX complex. MDCK II cells treated with 10 µg of ETX for 60 min at 4°C or 37°C. After washing and incubation with 10% SDS for 30 min, the samples were electrophoresed on an 8% polyacrylamide gel containing 10% SDS. Arrows depict, as specified, migration of the ∼155 kDa ETX complex or the ∼30 kDa ETX monomer. B) Quantification of ETX complex stability in the presence of 10% SDS at 4°C or 37°C. Fluorescence analysis of the ETX complex shows greater disruption by 10% SDS at 4°C than at 37°C, supporting temperature-sensitive conformational differences in the complex. Shown are the average results from 3 experiments.

Petit et al. reported [2] that, after solubilization, the ETX complex formed in MDCK II cells at 4°C appeared to be less stable in the presence of 10% SDS than is the ETX complex formed at 37°C [2]. That observation could suggest the existence of conformational differences, perhaps more subtle than can be picked up by the pronase digestion susceptibility assay, between the ETX complexes formed at 4°C vs. 37°C. The current study confirmed that observation by Petit et al. and then quantified, in a head-to-head comparison, the extent of this effect. Starting with the same amount of ETX complex present in MDCK II cells that had been treated with AF488-ETX at 4°C or 37°C (Fig. 8A), those complexes were extracted from cells using 10% SDS and then electrophoresed. Fluorescence scanning determined that 54.9% less of the AF488-ETX complex formed at 4°C remains intact under these high SDS conditions compared against the ETX complex formed at 37°C (Fig. 8B). These differences in ETX complex stability in the presence of high concentrations of SDS could reflect a conformational change that develops in the ETX complex at 37°C when it inserts into membranes and forms a stable pore.

## Discussion

Several previous studies have reported that ETX is not cytotoxic for sensitive cell lines, such as MDCK II cells, at 4°C [2], [21], [30], [41]. However, no mechanistic explanation for this observation had yet been offered. By investigating this topic, the current study has generated evidence supporting a new step in ETX action, i.e., formation of a prepore ETX oligomer on the membrane surface prior to insertion of that ETX complex into membranes to form the active pore.

At the start of this study, it remained possible that ETX binding or complex formation in MDCK II cells might be influenced by temperature. Petit et al. had previously reported [2] that ETX can bind and form the ETX complex required for cytotoxicity at 4°C but they did not perform a quantitative comparison of these two processes at 4°C vs. 37°C. In contrast, Soler-Jover et al. had determined that an ETX-GFP fusion protein, which is cytotoxic at 37°C but not at 4°C, binds in a temperature-dependent process [30]. For example, they showed that MDCK II cells treated with the fusion protein at 4°C, washed and then shifted to 37°C failed to develop any cytotoxicity [30]. When our current study repeated that experiment using native ETX, cytotoxicity developed at levels near those of a similar constant ETX treatment at 37°C. Furthermore, both toxin binding and complex formation were clearly observed at 4°C, with quantitative analyses using AF-labeled ETX indicating that these two early steps in ETX action develop to a similar extent at 4°C and 37°C. One possible explanation for the binding differences observed by Soler-Jover et al. [30] vs. those of our study and the study by Petit et al. [2] could be that the presence of a GFP moiety might alter the binding properties of the ETX-GFP fusion protein used by Soler-Jover et al., resulting in impaired binding and complex formation at 4°C.

Since the first two steps in native ETX action, i.e., binding and complex formation, were not significantly reduced at 4°C, the current study next examined the temperature dependence of the other previously recognized step in ETX action, i.e., pore formation. ETX was already known to cause a rapid and massive potassium release from MDCK II cells at 37°C [2], [21], [26], which the current study confirmed by demonstrating that, at 37°C, MDCK II cells show a strong ETX-induced release of cytoplasmic ^86^Rb, a potassium transport analogue. More importantly, despite the ability of ETX to bind and form a complex in MDCK II cells at 4°C, the current study found those cells exhibit no ETX-induced ^86^Rb release. To our knowledge, this is the first evidence that development of a functional ETX pore is blocked at 4°C, which explains why no ETX-induced cytotoxicity occurs at low temperature.

Pore formation by several other PFTs is also blocked at low temperature [28], [37], [42], [43]. However, the low temperature-sensitive step responsible for the loss of pore formation varies amongst different PFTs. In general, PFT binding tends to be temperature-independent, occurring equally well at 4°C and 37°C; however, there are exceptions, e.g., *C. perfringens* enterotoxin causes less cytotoxicity at 4°C, in part, because of reduced receptor binding of this toxin at low temperature [27], [29], [37]. The pore-forming ability of some other PFTs, e.g. *Vibrio cholerae* cytolysin, is reportedly inhibited at 4°C because their oligomerization is temperature-sensitive [43]. In addition to impaired receptor binding at 4°C, the oligomerization of *C. perfringens* enterotoxin is also temperature-sensitive [27], illustrating that low temperatures can sometimes interfere with several early steps in the action of a pore-forming toxin. The current findings indicate that ETX differs from such PFTs in that it can bind and form oligomeric complexes equally well at both 4°C and 37°C.

Oligomerization of several PFTs occurs on the membrane surface, resulting in a prepore stage prior to membrane insertion of the toxin oligomer to form an active pore [28], [37], [42]. The most important results of the current study are the first direct experimental evidence supporting the oligomerization of ETX in a prepore stage on the membrane surface. First, the current study demonstrated that, when MDCK II cells are treated with ETX at 4°C and then shifted to 37°C, they exhibited almost similar levels of cytotoxicity as occurs when those cells are constantly treated with ETX at 37°C. This result is consistent with the ETX complex formed at 4°C representing a prepore intermediate that can, under permissive conditions, become a functional ETX pore. Second, this work showed that, when solubilized from MDCK II cells, the ETX complexes formed at 4°C or 37°C are equally susceptible to pronase degradation; however, when present in intact MDCK II cells, the ETX complex formed at 4°C is much more susceptible to pronase degradation than is the ETX complex formed at 37°C. Since the ETX complex at 37°C has inserted into membranes to form an active pore, the temperature-related differences in pronase susceptibility of ETX complexes in intact cells provide evidence that the ETX complex formed at 4°C remains exposed on the membrane surface in a prepore. Finally, the ETX complex formed at 4°C was shown to dissociate more readily from MDCK II cells compared against the ETX complex formed in MDCK II cells at 37°C. This result is consistent with i) the complex formed at 37°C being trapped in membranes due to its insertion to form an active pore but, ii) the complex formed at 4°C more easily dissociating from cells because it remains surface-localized in a prepore. Collectively, these results offer experimental support for the earlier hypothesis of Pelish and McClain that ETX might be a PFT that forms a pre-pore intermediate prior to active pore formation [20].

This new evidence for a prepore stage in ETX action highlights the similarity in action between ETX and *C. septicum* alpha toxin [28]. *C. septicum* alpha toxin resembles ETX by exhibiting temperature-independent binding and oligomerization, yet temperature-sensitive pore formation. In addition, Sellman et al. reported evidence indicating that *C. septicum* alpha toxin has a temperature-sensitive prepore assembly step that occurs prior to its insertion into membranes to form an active pore [28]. This similarity between the actions of ETX and *C. septicum* alpha toxin is notable since alpha toxin shares sequence homology with aerolysin and aerolysin possesses structural similarity to ETX (the structure of *C. septicum* alpha toxin has not yet been solved) [10], [44]. However, it is interesting that, while aerolysin can bind and oligomerize at low temperatures [45], a distinct prepore step in the action of that PFT has not yet been observed [45]. Instead, it has been proposed that oligomerization and membrane insertion of aerolysin may be concomitant events [45].

Future studies are needed to determine precisely why, at 4°C, ETX remains surface-localized in a prepore. However, we did observe a correlation between formation of the ETX pore and temperature, with ETX-treated MDCK II cells exhibiting no ^86^Rb-release at 4°C (Fig. 5), intermediate levels of ^86^Rb-release at 20°C (data not shown), and massive ^86^Rb-release at 37°C (Fig. 5). This result could indicate that ETX pore formation in MDCK II cells is influenced by membrane fluidity. That possibility would be consistent with conclusions about ETX insertion drawn from earlier studies examining ETX action on liposomes [46]. However, comparisons between results using MDCK II cells and liposomes should be made cautiously since liposomes are 100-fold less sensitive to ETX compared against MDCK II cells and, at 4°C, ETX binding and oligomerization are also inhibited in liposomes, a situation unlike what has been observed in MDCK II cells (this study; [2]).

Petit et al. observed that the ETX complex formed at 4°C is much less stable in the presence of 10% SDS compared against the ETX complex formed at 37°C [2]. This observation was confirmed in the current study. Given the new information supporting the trapping of ETX in a prepore stage at 4°C, these differences in SDS stability of ETX complexes at 4°C vs. 37°C could reflect temperature-related conformational differences due to the presence of ETX complex in a prepore vs. active pore state.

**Figure 9:**
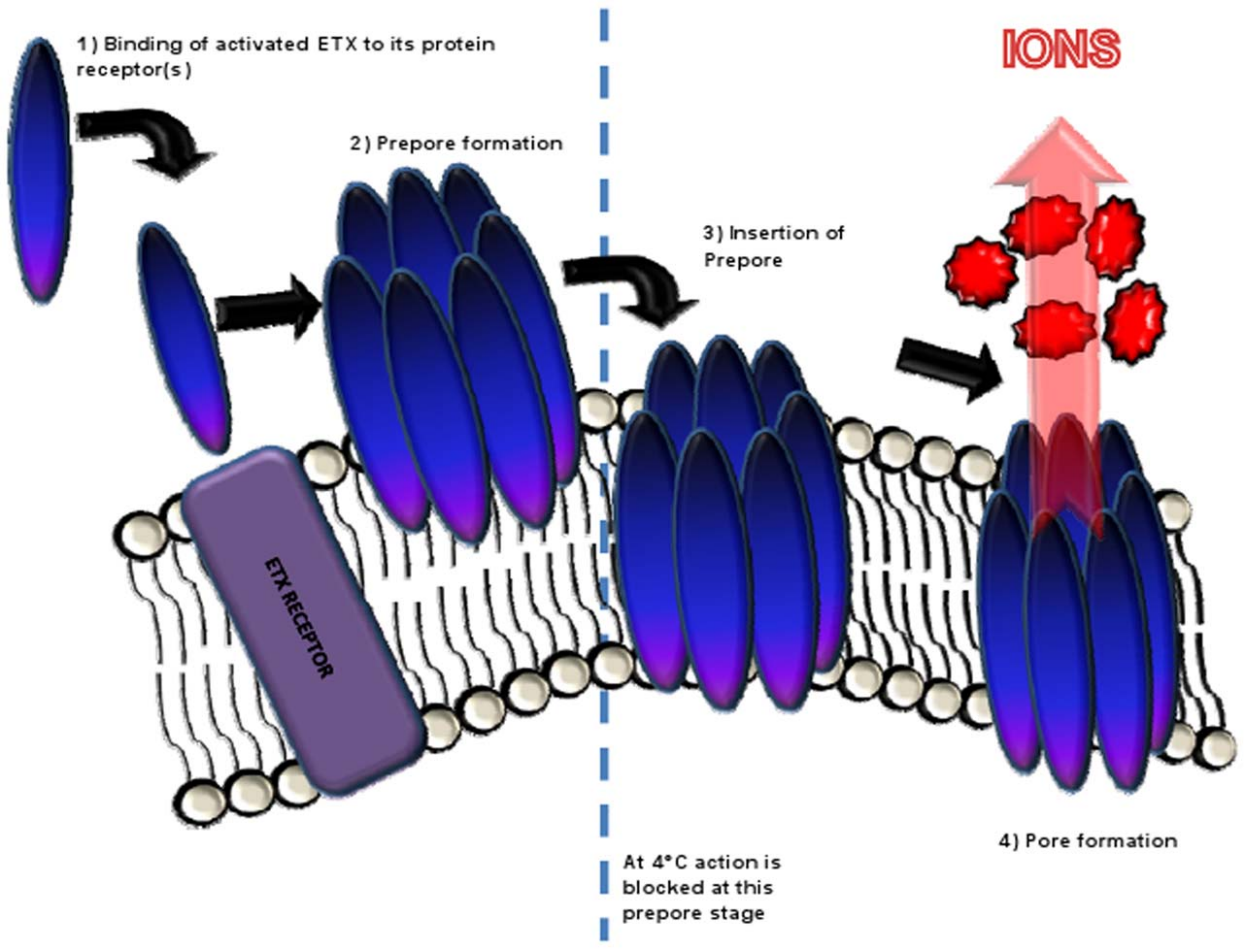
Updated Model for ETX action in MDCK II cells. Mode of action begins with activated ETX binding to proteinacous membrane receptor. Bound ETX then oligomerizes to form a heptameric prepore on the membrane surface. At 4°C, the process stops here, but at 37°C the prepore intermediate inserts into the membrane to complete pore formation.

In summary, results from the current study update our understanding of ETX action to fit the model shown in Fig. 9. ETX binds to an uncharacterized receptor and then oligomerizes to form a prepore intermediate on the membrane surface. At 4°C, the process stops here, possibly because of limited membrane fluidity (although this hypothesis requires further study). At 37°C, the prepore rapidly undergoes a conformational change to insert into membranes and form the active pore. This pore formation then triggers efflux of certain cytoplasmic ions such as potassium and influx of other ions such as calcium. These pore-induced permeability effects cause or contribute to ETX-induced cell death.
